# Enhanced Bone Tissue Regeneration by Porous Gelatin Composites Loaded with the Chinese Herbal Decoction Danggui Buxue Tang

**DOI:** 10.1371/journal.pone.0131999

**Published:** 2015-06-30

**Authors:** Wen-Ling Wang, Shi-Yuan Sheu, Yueh-Sheng Chen, Shung-Te Kao, Yuan-Tsung Fu, Tzong-Fu Kuo, Kuo-Yu Chen, Chun-Hsu Yao

**Affiliations:** 1 School of Chinese Medicine, China Medical University, Taichung, Taiwan; 2 Department of Chinese Internal Medicine, China Medical University Hospital, Taichung, Taiwan; 3 School of Medicine, Chung Shan Medical University, Taichung, Taiwan; 4 Department of Integrated Chinese and Western Medicine, Chung Shan Medical University Hospital, Taichung, Taiwan; 5 Department of Biomedical Imaging and Radiological Science, China Medical University, Taichung, Taiwan; 6 Department of Chinese Medicine, Taichung Tzu Chi Hospital, The Buddhist Tzu Chi Medical Foundation, Taichung, Taiwan; 7 Department of Veterinary Medicine, School of Veterinary Medicine, National Taiwan University, Taipei, Taiwan; 8 Department of Chemical and Materials Engineering, National Yunlin University of Science and Technology, Yunlin, Taiwan; 9 Department of Biomedical Informatics, Asia University, Taichung, Taiwan; Chang Gung University, TAIWAN

## Abstract

Danggui Buxue Tang (DBT) is a traditional Chinese herbal decoction containing Radix Astragali and Radix Angelicae sinensis. Pharmacological results indicate that DBT can stimulate bone cell proliferation and differentiation. The aim of the study was to investigate the efficacy of adding DBT to bone substitutes on bone regeneration following bone injury. DBT was incorporated into porous composites (GGT) made from genipin-crosslinked gelatin and β-triclacium phosphates as bone substitutes (GGTDBT). The biological response of mouse calvarial bone to these composites was evaluated by *in vivo* imaging systems (IVIS), micro-computed tomography (micro-CT), and histology analysis. IVIS images revealed a stronger fluorescent signal in GGTDBT-treated defect than in GGT-treated defect at 8 weeks after implantation. Micro-CT analysis demonstrated that the level of repair from week 4 to 8 increased from 42.1% to 71.2% at the sites treated with GGTDBT, while that increased from 33.2% to 54.1% at GGT-treated sites. These findings suggest that the GGTDBT stimulates the innate regenerative capacity of bone, supporting their use in bone tissue regeneration.

## Introduction

It is urgent to develop novel methods for accelerating the repair and restoration of large bone defects [1]. Biodegradable composites based on synthetic bone-promoting biomaterials have been widely investigated for use in bone tissue engineering and bone regeneration [2–4]. Bioactive ceramics, such as tricalcium phosphate, hydroxyapatite, and bioglass, have been utilized as hard tissue implant materials due to their excellent bone conductivity [5–7]. Natural polymers, such as polypeptides (collagen and gelatin) and modified polysaccharides (chitosan), and synthetic polymers, such as poly(glycolic acid) and poly(L-lactic acid), have been previously used as binders with desired adhesive and plastic properties and favorable biocompatibility [4,8,9].

Our previous work developed a bone substitute (GGT) consisted of genipin cross-linked gelatin mixed with β-tricalcium phosphate (β-TCP) ceramic particles. The results demonstrated that the gelatin molecules and calcium ions gradually released from the composite enhanced the proliferation and differentiation of the osteoblasts *in vitro* [10]. Furthermore, *in vivo* studies have shown that the GGT composite has adequate biodegradability and biocompatibility, and enhances new bone growth into the calvarial bone defect [11,12]. Moreover, a gelatin-based scaffold can act as a carrier for delivering osteoinductive agents to promote the repair of bone defects [13].

Traditional Chinese herbal medicines have been extensively used to stimulate the healing of bone fractures and bone defects [14]. Among these herbal medicines, Danggui Buxue Tang (DBT), a formula consisting of Radix Angelicae sinensis and Radix Astragali, has been widely used to improve hematopoietic function, stimulate cardiovascular circulation, prevent osteoporosis, promote anti-oxidation activity, and both stimulate and modulate immune functions [15,16]. Research on Radix Angelicae sinensis has established that it induce the proliferation, alkaline phosphatase activity and type I collagen synthesis of human osteoprecursor cells *in vitro* [17]. They have also been demonstrated to increase the proliferation and differentiation of human osteosarcoma cell line MG-63 [18].

Our earlier work showed that DBT has an osteotropic effect, which is strongest at a Radix Astragali to Radix Angelicae sinensis ratio of 5:1. The optimal concentration of DBT is 1,000 μg/mL, which markedly increases the number of osteoblasts, intracellular alkaline phosphatase levels, and number of bone nodules, while decreasing osteoclast activity [19]. More investigations of the effectiveness of adding Chinese herbal medicine to bone substitutes are conducted [20–22]. Via an effective biodegradation delivery system, the released DBT is expected to promote the growth of bone tissue.

In this study, a mixture of GGT composite and DBT extracts (GGTDBT) was fabricated to fill the mouse calvarial defect to evaluate the effect of DBT on bone formation *in vivo*. Bone repair was assessed by *in vivo* imaging system (IVIS), micro-computed tomography (micro-CT), and histological examination.

## Materials and Methods

### Preparation of DBT Solution and Porous Composites

The Chinese herbal decoction DBT utilized herein comprised Huangqi (Radix Astragali) and Danggui (Radix Angelicae sinensis) in a weight ratio of 5:1. Fresh roots were obtained from Chuang Song Zong Pharmaceutial Co., Ltd. (Kaohsiung, Taiwan). Aqueous DBT extracts were prepared in standardized procedures [19]. Briefly, Radix Astragali and Radix Angelicae sinensis were boiled separately in six times volume of water for 1 h. The residue from the first extraction was boiled in eight times volume of water for another 1.5 h. The filtered solutions were then combined and passed through a 0.22 μm Millipore filter. The filtrates were frozen at –20°C until use.

GGT composites were prepared as described previously [11]. Briefly, porcine gelatin (Bloom number 300, Sigma-Aldrich, St. Louis, MO) was dissolved in deionized water at 70°C to obtain an 18 wt% solution. When the gelatin solution was cooled to 40°C, 0.5 wt% of genipin solution (Challenge Bioproducts Co., Yunlin, Taiwan) was added to induce crosslinking reaction. After stirring at 40°C for 2 min of, β-TCP ceramic particles (Merck, Darmstadt, Germany) with grain sizes of 200–300 μm and sieved sodium chloride particles with sizes 250–470 μm were mixed vigorously to make the solution viscous. The weight ratios of gelatin to β-TCP and sodium chloride to gelatin/β-TCP/genipin mixture were 1: 3 and 3: 1, respectively. The mixture was poured into plastic dishes to solidify. The solidified composites were then immersed into deionized water for 24 h to leach out the sodium chloride porogens. The samples were frozen at –80°C for 24 h, and then lyophilized in a freeze dryer for another 24 h to form porous GGT composites. The GGTDBT composites were prepared by a similar procedure as described above, expect that gelatin was dissolved in 1,000 μg/mL of DBT solution instead of in deionized water. The weight ratio of DBT to gelatin to β-TCP in the GGTDBT composite was approximately 0.1:18:54.

### Biocompatibility of GGT and GGTDBT Composites

The *in vivo* biocompatibility of GGT and GGTDBT composites were conducted via subcutaneous implantation in rats. 8 adult male Sprague-Dawley rats, purchased from the National Laboratory Animal Center, Taiwan, with an average weight of 280–300 g were used. All animals were placed in temperature-controlled (23°C) and humidity-controlled (45%) rooms with 12-h dark-light cycles and were given food and water *ad libitum*. All animal procedures were performed with the approval of the Institutional Animal Care and Use Committee of China Medical University. The rats were anaesthetized by intramuscular injection of Zoletil 50 (Virbac Laboratories, Carros, France) and 2% Rompun (Bayer, Leverkusen, Germany) (1:2 ratio, 1 mL/kg). The dorsal surface of rat was shaved and sterilized with povidone iodine solution (Sinphar Pharmaceutical Co., Yilan, Taiwan). Skin incisions approximately 5 mm in length were made on the dorsal surface of each rat to insert the GGT and GGTDBT composites. After 4 and 8 weeks of implantation, rats were sacrificed and the tissue-covered implants were prepared for histological observations. The samples were sectioned, stained with hematoxylin-and-eosin (H&E), and examined by an optical microscope (Olympus IX70, Japan).

### *In vivo* Calvarial Defects in Mice

Experimental cranial implantation was performed on 24 ICR strain mice (6 weeks old; from the National Laboratory Animal Center, Taiwan). The mice were anaesthetized with an intramuscular injection of Zoletil 50 and 2% Rompun (1:2 ratio, 0.4 mL/kg). The head of the animal was shaved and disinfected with povidone iodine solution. Midline incision was made to expose the skull. The overlying parietal periosteum was then excised. A 5 mm diameter circular defect was created in the cranium using a high-speed drill with a cutting bur taking care to avoid to damage the superior sagittal sinus or to interrupt the dura mater under the bone. The calvarial bone defects were filled with sterile GGT and GGTDBT composites to evaluate their bone-regenerative capability. Bone healing was observed by IVIS (Xenogen Alameda, CA), micro-CT (SkyScan-1176, Aartselaar, Belgium), and histology analysis.

IVIS analysis was performed on 12 mice. 6 mice were examined at each time point. At 4 and 8 weeks post-surgery, the animal was anaesthetized and injected via tail vein with 100 μL of RediJect Bone Probe 680 (concentration; 2 nmols/150 μL; Caliper Life Science, Inc., Hopkinton, MA). One hour later, animals were placed faced down in the chamber and imaged for 5 min using an IVIS imaging system 200 series. The intensities of fluorescence emitted by the fluorophores were quantified using Living Image software (Xenogen). Data are presented as mean fluorescence intensity (MFI ± SD) within the region of interest (ROI) applied at calvarial defect sites.

The anesthetized animals were sacrificed by injection of an overdose of sodium pentobarbitol at 4 and 8 weeks postoperatively. 6 mice were sacrificed at each time point. Both the radiographical and histological analyses were conducted for each animal. The crania were removed and cleaned. Specimens were submerged in a 10% neutral formalin-buffered solution (Merck, Whitehouse Station, NJ), which was changed once after 24 h to ensure good fixation. The micro-CT images were obtained using a commercially available micro-CT scanner and the newly formed bone was quantified by counting the number of bone voxels using ImageJ software (National Institutes of Health, Bethesda, MD). The regions of interest were constructed from multiple tomography slices for both cortical and trabecular bones.

The regeneration of defective bones was also analyzed histologically. After 4 and 8 weeks of operation, the animals were anesthetized and sacrificed in a carbon dioxide-filled box. The craniectomy sites were removed, decalcified, formalin-fixed, alcohol-dehydrated, xylene-cleared, paraffin-embedded, and then stained with H&E. The stained sections were observed using an inverted optical microscope (Axiovert 25, Carl Zeiss, Göttingen, Germany).

### Statistical Analysis

Numerical data were presented as mean ± standard derivation. Statistical analysis was performed using one-way analysis of variance (ANOVA) followed by post hoc Fisher's LSD multiple comparison test. The levels of statistical significance were set to *p* < 0.05.

## Results

### *In vivo* Evaluation of Biocompatibility by Subcutaneous Implantation

**Fig 1:**
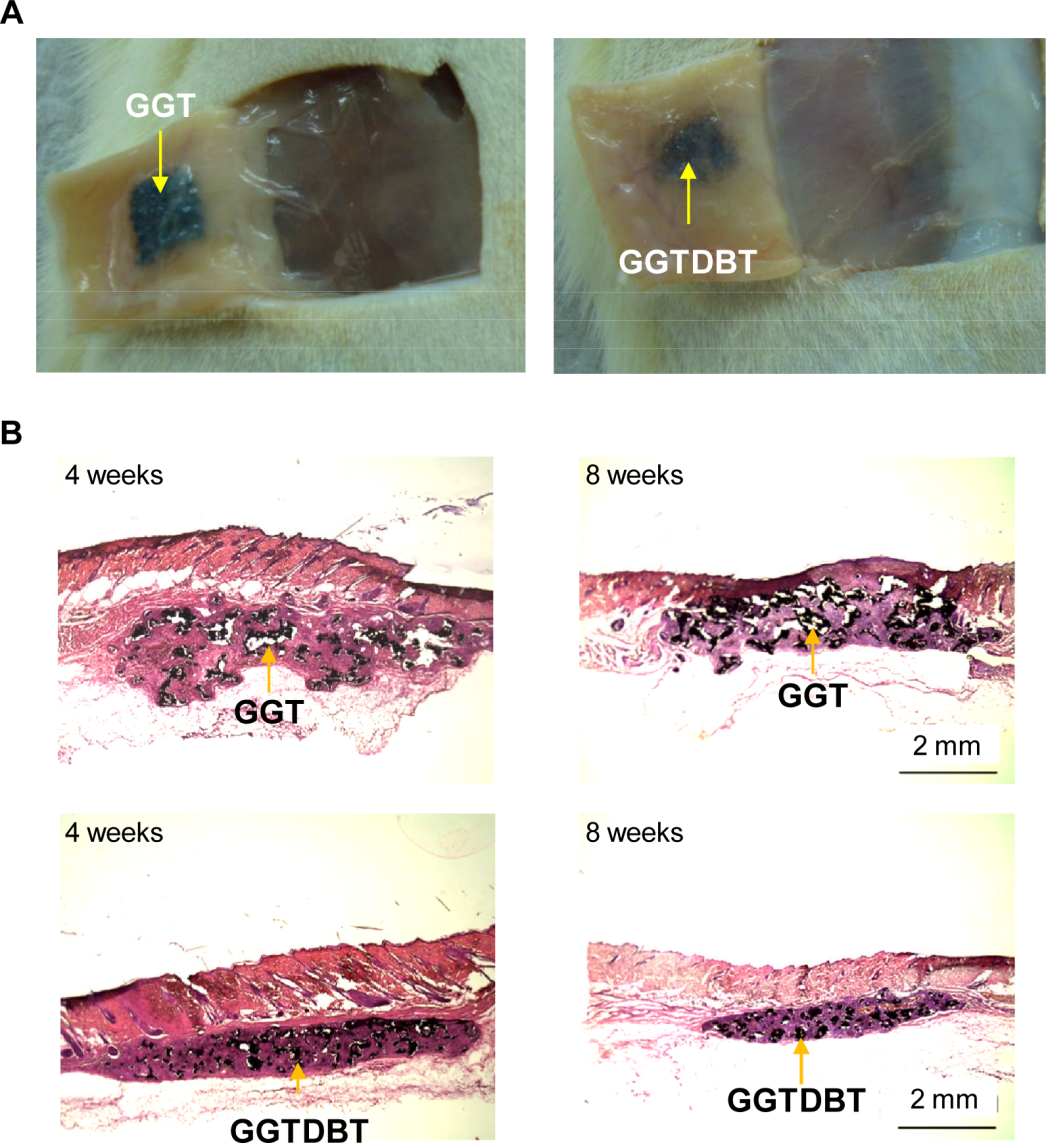
Biocompatibility of composites. (A) Macroscopic observations of tissue-covered implants, after GGT and GGTDBT composites were implanted subcutaneously at 8 weeks post-surgery. (B) Tissue reaction after GGT and GGTDBT composites were implanted for 4 and 8 weeks.

No complication, such as wound infection, inflammation, festering, hematoma, or disturbed wound healing at the surgical sites, was found for any of the rats throughout the experiments, as shown in Fig 1A. The histological changes in tissue specimens were examined by the H&E staining at 4 and 8 weeks post-surgery (Fig 1B). The GGT and GGTDBT composites did not result in histopathological changes, indicating that these composites exhibited favorable biocompatibility.

### Biological Response of Mouse Calvarial Bone

**Fig 2:**
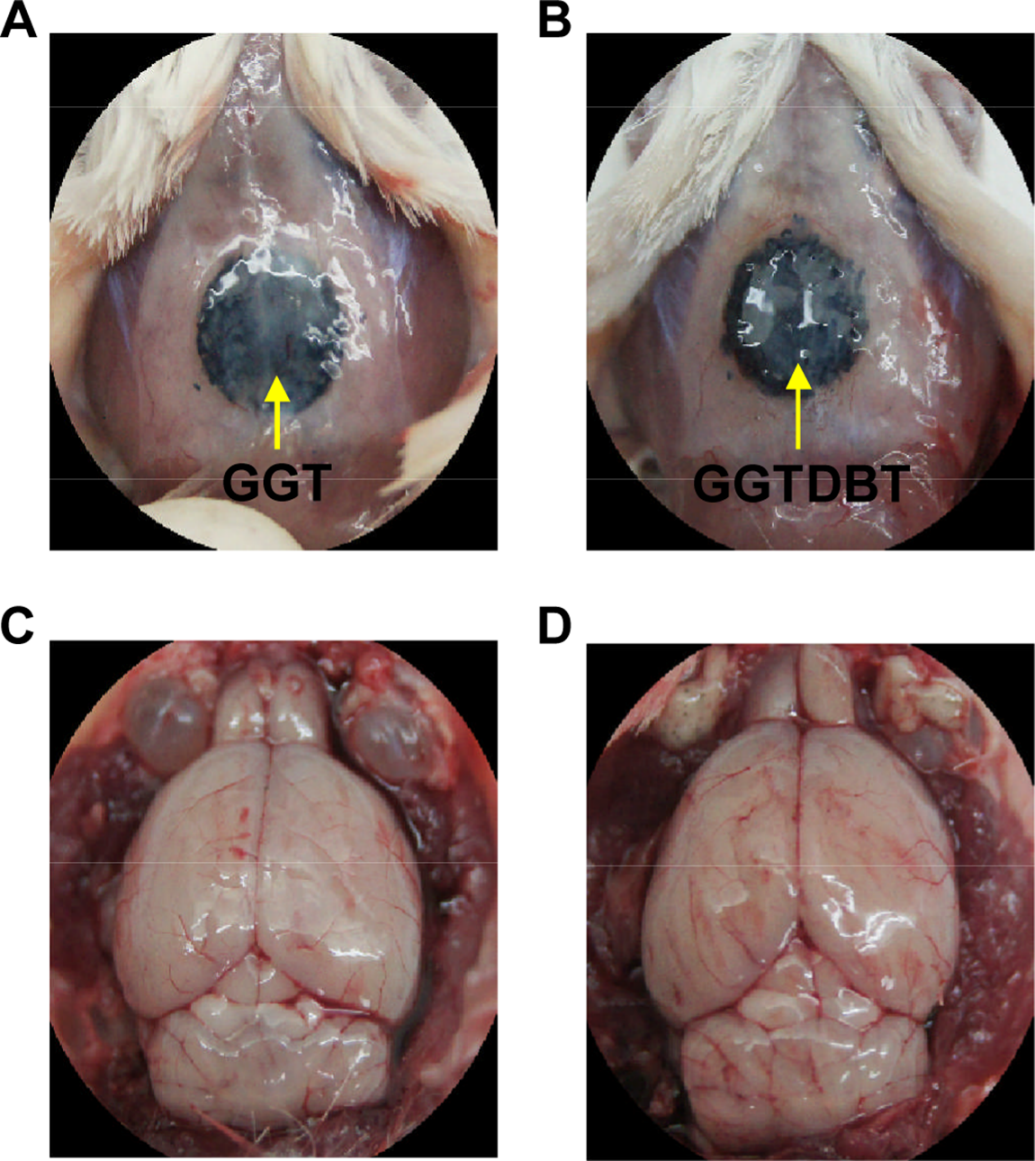
Gross observations of calvarial bone defect sites after 8 weeks treatment. No evidence of infection, inflammation, hematoma, or necrosis was observed at the implant sites around the calvarial bone defects treated with (A) GGT and (B) GGTDBT composites. The brain tissues under the implantation sites exhibited no evidence of adverse tissue reaction to the (C) GGT and (D) GGTDBT composites.

All animals survived for the duration of the study (8 weeks) without clinical complications. There was no sign of infection, inflammation, hematoma, or necrosis at the site of implants. Closure of the cortical window and uniformly filling the borders of the defects with new bone were seen at the defect sites treated with the GGT and GGTDBT composites (Fig 2A and 2B). Moreover, the brain tissues under the implantation sites exhibited no evidence of cortical inflammation, scar formation, or an adverse tissue reaction to the GGT or GGTDBT composites, suggesting that the substances released from the composites did not harm the underlying brain tissues (Fig 2C and 2D).

**Fig 3:**
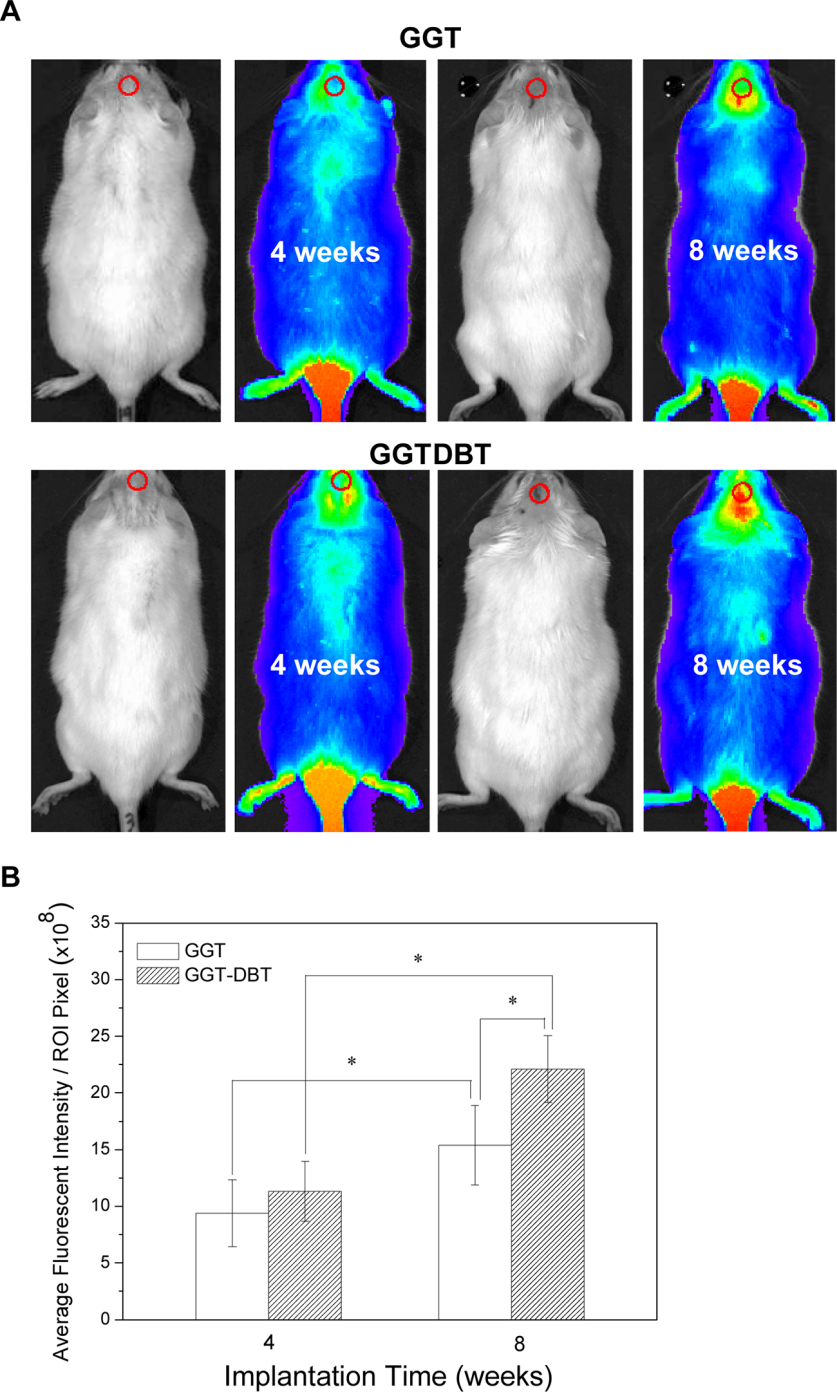
Bioluminescence images. (A) IVIS images of bioluminescent GGT and GGTDBT composites at 4 and 8 weeks postimplantation into rat calvarial defects. (B) Mean fluorescence intensity by region of interest in defects, as measured by IVIS imaging at 4 and 8 weeks. Bone healing proceeds over the study period. * *p* < 0.05.

The GGT and GGTDBT composites were implanted into calvaria defects and subjected to IVIS analysis at 4 and 8 weeks postoperatively (Fig 3A). Fluorophore specific for hydroxyapatite of bone was used to visualize new bone formation without sacrificing the animals. Each group exhibited a higher level of bioluminescence at week 8 than at week 4 (*p* < 0.05) (Fig 3B). Quantification analysis also demonstrated that adding DBT increased the amount of new bone tissue over that in the GGT group at week 8 (*p* < 0.05).

**Fig 4:**
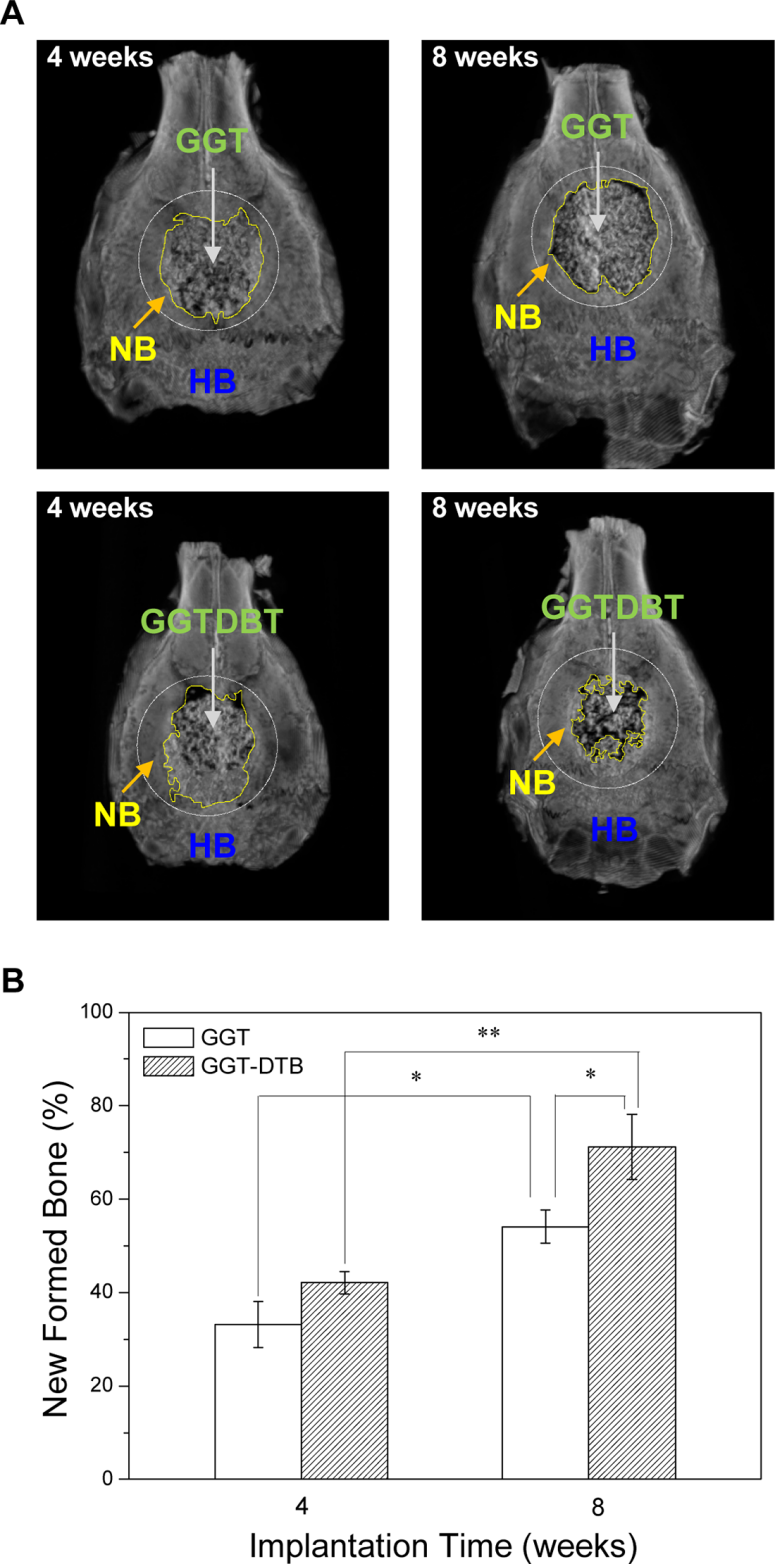
Micro-CT images. (A) Micro-CT images of calvarial bone during regeneration over study period. Progressive wound healing is observed, and continuity of newly formed bone network at GGTDBT and GGT-treated sites is comparable to that of host bone in weeks 4 and 8. (HB = host bone, NB = new bone, and area in *circular margin* indicates original margin of calvarial bone defect. (B) Percentage area repair rate in defects, as measured by micro-CT at 4 and 8 weeks. Amount of regenerated bone increased over study period. * *p* < 0.05, **p < 0.01.

Microstructural data were available by micro-CT image analysis at 4 and 8 weeks following the implantation of the GGT and GGTDBT composites. Fig 4A displays that GGT and GGTDBT were well integrated into the surrounding calvarial bone and new bone was formed at the interface between the host bone and the implants, indicating good tissue compatibility and osteoconduction of both composites. Moreover, there was more extensive new bone formation in the GGTDBT than in the GGT at the same period of implantation. Additionally, the new bone formation became more prominent as the implantation time increased. Fig 4B presents the amount of newly formed bone as a percentage of the volume of calvarial bone defect in each implantation-examination period. The percentage of bone repair significantly increased with the implantation period (*p* < 0.05). A notable increase in percentage of bone repair from 42.1% to 71.2% at the sites treated with GGTDBT from week 4 to week 8 was observed when compared to that from 33.2% to 54.1% at the sites treated with GGT. These results suggest that DBT released from the degraded composite enhanced bone defect repair.

**Fig 5:**
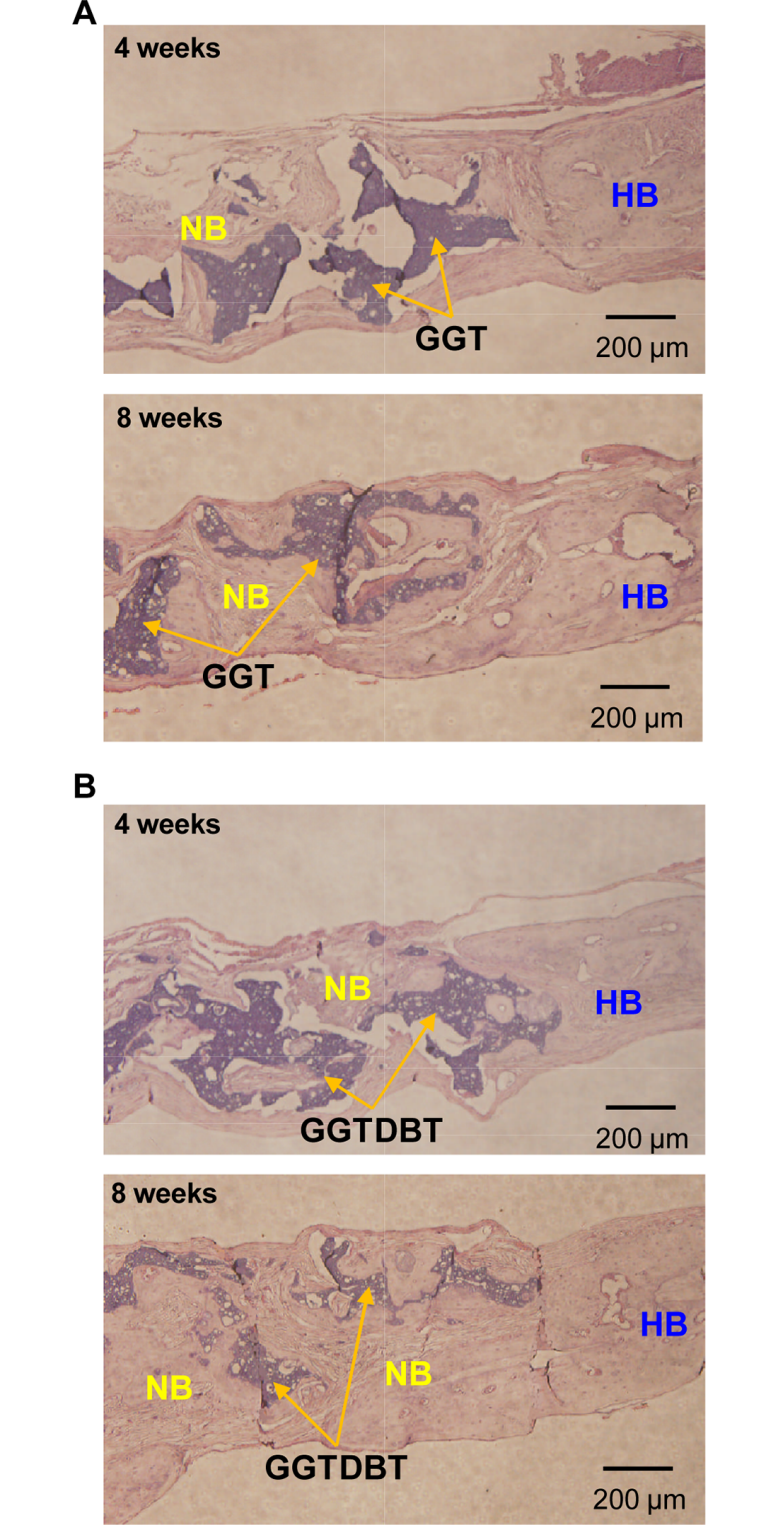
Histological image of H&E-stained (A) GGT and (B) GGTDBT composites, implanted in calvarial bone defects for 4 and 8 weeks. Regeneration of bone continued for weeks after implantation; newly formed bone replaced more GGTDBT composite than GGT composite. (HB = host bone; NB = new bone).

Finally, a histological analysis was performed to assess the osteogenicity of the GGT and GGTDBT composites. The histopathological observation reveals the progressive growth of the new bone tissue into the calvarium defect (Fig 5). At 4 weeks postoperatively, a thin layer of new bone was observed adjacent to the host bone tissue, confirming the osteoconductive nature of the material. At week 8, new bone was also observed growing into the interior of the defect by replacing significant amounts of the composite. The newly formed bone began connected and tended to grow across the defect. As characterized histopathologically, GGTDBT produced more new bone tissue than was obtained at the GGT-treated sites. Moreover, markedly more new bone was formed in the calvarial bone defects treated with the GGTDBT 8 weeks after implantation than was present after 4 weeks of implantation. The healing was uniform and the healing rate was faster at all GGTDBT composites-treated sites than at the GGT composites-treated sites.

## Discussion

Bone replacement materials should exhibit favorable biocompatibility, biodegradability, and regenerative characteristics (including osteoinduction, osteoconductivity, and osteogenesis), to conduct cells to defect sites and promote new bone formation. In the authors’ earlier feasibility studies, composite scaffold material, such as GGT, is biodegradable and has good biocompatibility to support cell attachment and growth [10,11]. Moreover, the composite was investigated in an attempt to cause osteoconduction to regenerate bone tissues [12]. Calcium phosphate in the composite tends to promote bone regeneration and the resorption of graft material in bone defects. When degradation occurs, soluble inorganic calcium and phosphate ions released from the composite provide an ideal setting for cell colonization, proliferation, and differentiation to form new bone *in vivo* [23]. Gelatin provides a platform for exploiting the plastic properties of scaffolds in tissue engineering [24].

In this work, a GGT composite combined genipin-crosslinked gelatin and β-TCP was used as a bone substitute. The weight ratio of the gelatin to β-TCP in the composite was 1/3 to mimic the organic/nonorganic ratio in natural bone [25]. As stated earlier, GGT exhibited osteoconductive properties rather than osteoinductive capabilities. An increasing number of studies of the effectiveness of adding a bone formation-inducing factor, such as Achyranthes bidentata Bl., Cuscuta chinensis Lam., Dipsacus asper Wall., Drynaria fortune, Eucommia ulmoides Oliv., Loranthus parasiticus Merr., Chi-Li-Saan, and naringin to a bone substitute have demonstrated that doing so accelerates the ingrowth of new bone into defective sites [21,22,26–28]. The properties of the above materials are critical for osteoinduction to occur.

Danggui Buxue Tang, a well-known Chinese herbal decoction with tonified Qi and a blood engendering function, is valuable in bone healing and regeneration [14]. The osteotropic effects of DBT extracts on the potential of enhancing bone formation via *in vitro* bone cell proliferation and differentiation have been studied elsewhere [18]. Our earlier investigation established that DBT clearly enhanced the proliferation and differentiation of osteosarcoma MG-63 cells and nodules formation [19]. 1,000 μg/mL of DBT considerably improved the osteogenic proliferation, differentiation, and mineralization of osteoblastic cells and inhibited the activity of osteoclasts. In the present work, an osteogenic GGT composite was extensively investigated, and 1,000 μg/mL of DBT extract was added in the preparation of GGTDBT composite with the expectation of promoting bone tissue regeneration *in vivo*.

The *in vivo* biocompatibilities of GGT and GGTDBT were evaluated following subcutaneous implantation in rats. The *in vivo* bone regenerative capacities of GGT and GGTDBT were investigated using a rat calvarial defect model. Bone defect repair was examined at 4 and 8 weeks postoperatively. Since both GGT and GGTDBT used herein were moldable and malleable, all experimental bone defects were perfectly filled by the two materials. Gross examination showed that GGT and GGTDBT were biocompatible. As the composites degraded, some of their constituents, such as calcium, phosphate, gelatin, and DBT, were released into defects, supplying nutrients for new bone formation.

After 4 and 8 weeks of implantation, IVIS analysis showed a much stronger bioluminescent signal detected in GGTDBT-treated sites than in GGT-treated sites. Micro-CT images demonstrated new bone growth into the calvarial defects. Moreover, the GGTDBT-treated sites had a higher repair rate than GGT-treated sites. The enhanced bone volume verified progressive defect healing, and the infiltration of new bone into the implant construct over time. Histological evaluations confirmed the osteoconductive process of the calvarial host bone, which was directed toward the bone defects, forming new bone close to the host bone-composite interface. More new bone was present in the calvarial bone defects treated with the GGTDBT composites after 8 weeks than was present after 4 weeks. According to the *in vitro* cell culture test, DBT promoted the proliferation and differentiation of osteoblastic cells and inhibited osteoclast activity [19]. This observation is consistent with the findings of recent studies, which have demonstrated that the GGTDBT construct stimulates the innate regenerative capacity of bone, and so underscores the use of this construct as valuable in bone tissue regeneration.

In conclusion, DBT-incorporating porous gelatin composite was utilized in this investigation to fill a bone defect in a calvarial model and it promoted bone regeneration with good osteoconductivie and osteoinductive potential. It also exhibited good biocompatibility and biodegradability. Hence, we claim that the GGTDBT construct stimulates the innate regenerative capacity of bone, and can be used in bone tissue regeneration.
